# Supplementary figures and images for: Effective and safe: Long-term aerosol disinfection of slightly acidic electrolyzed water causes no harm in rats
Source: PLoS One. 2026 Jan 30;21(1):e0341050. doi: 10.1371/journal.pone.0341050 (PMC12857984; doi:10.1371/journal.pone.0341050)

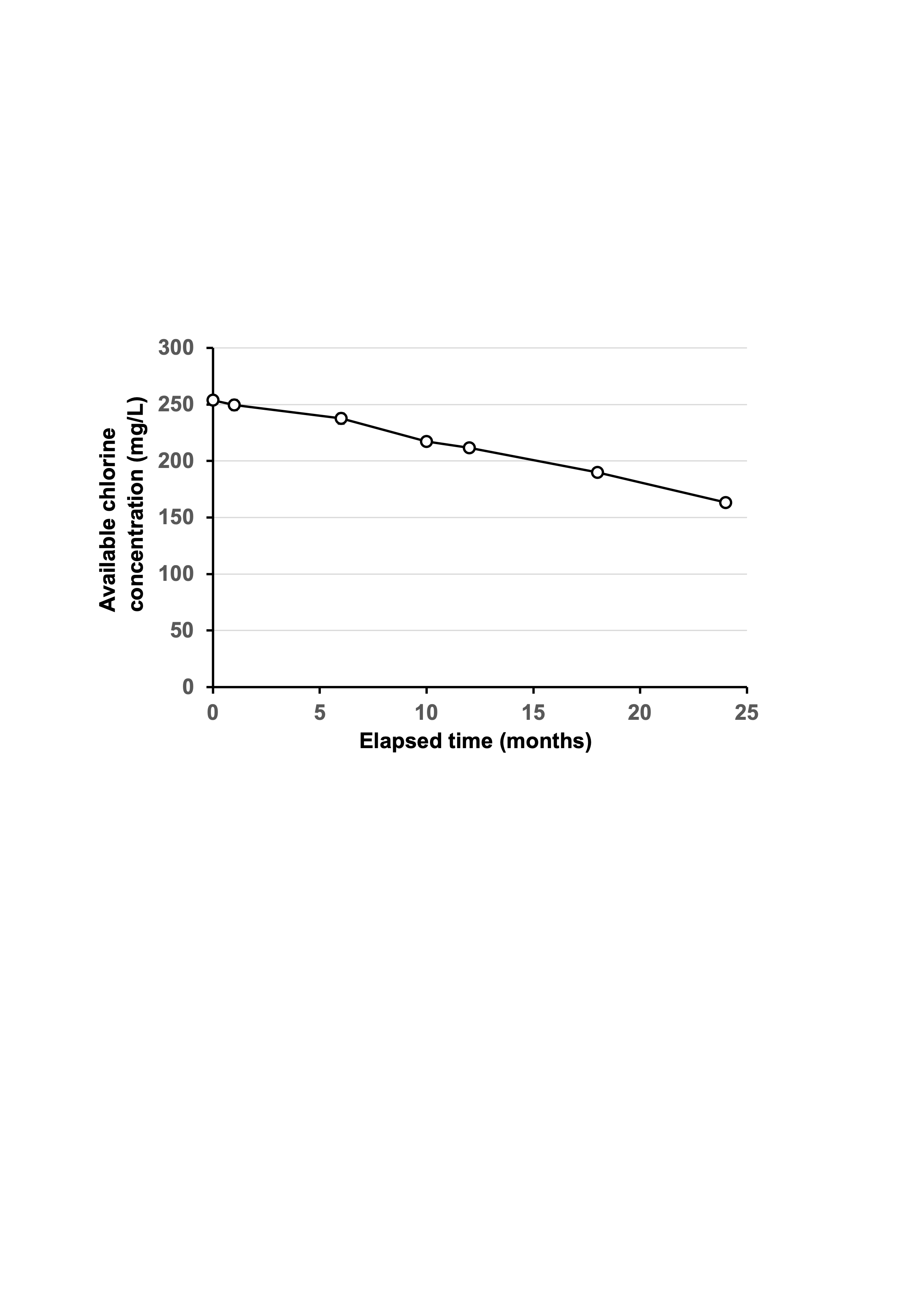

Supplement: S1 Fig — Data represent the mean ± standard error of three independent experiments. The standard errors were too small to be visible and are therefore obscured by the markers. (TIFF) [file pone.0341050.s001.tiff]
